# The Comet Assay in Drosophila: A Tool to Study Interactions between DNA Repair Systems in DNA Damage Responses In Vivo and Ex Vivo

**DOI:** 10.3390/cells12151979

**Published:** 2023-07-31

**Authors:** Rubén Rodríguez, Isabel Gaivão, Leticia Aguado, Marta Espina, Jorge García, Pablo Martínez-Camblor, L. María Sierra

**Affiliations:** 1 Department of Functional Biology (Genetic Area), University of Oviedo, 33006 Oviedo, Spain; ruben.rodriguez@evolutioneurope.eu (R.R.);; 2 Oncology University Institute from Asturias (IUOPA), University of Oviedo, 33006 Oviedo, Spain; 3 Department of Genetics and Biotechnology and CECAV, University of Trás-os-Montes and Alto Douro, 5000-801 Vila Real, Portugal; igaivao@utad.pt; 4 Department of Biomedical Data Science, Geisel Medical School at Dartmouth, Hanover, NH 03755, USA; pablo.martinez.camblor@dartmouth.edu; 5 Faculty of Health Sciences, Universidad Autónoma de Chile, Provedencia 7500912, Chile; 6 Institute of Sanitary Research of the Principality of Asturias, Av. del Hospital Universitario, s/n, 33011 Oviedo, Spain

**Keywords:** in vivo comet assay, ex vivo comet assay, DNA damage response, *Drosophila melanogaster*, NER, dmPolQ, *mus308*, *mus201*, *mus201;mus308* and *OK* strains, MMS

## Abstract

The comet assay in Drosophila has been used in the last few years to study DNA damage responses (DDR) in different repair-mutant strains and to compare them to analyze DNA repair. We have used this approach to study interactions between DNA repair pathways in vivo. Additionally, we have implemented an ex vivo comet assay, in which nucleoids from treated and untreated cells were incubated ex vivo with cell-free protein extracts from individuals with distinct repair capacities. Four strains were used: wild-type OregonK (*OK*), nucleotide excision repair mutant *mus201*, dmPolQ protein mutant *mus308*, and the double mutant *mus201;mus308*. Methyl methanesulfonate (MMS) was used as a genotoxic agent. Both approaches were performed with neuroblasts from third-instar larvae; they detected the effects of the NER and dmPolQ pathways on the DDR to MMS and that they act additively in this response. Additionally, the ex vivo approach quantified that *mus201*, *mus308*, and the double mutant *mus201;mus308* strains presented, respectively, 21.5%, 52.9%, and 14.8% of *OK* strain activity over MMS-induced damage. Considering the homology between mammals and Drosophila in repair pathways, the detected additive effect might be extrapolated even to humans, demonstrating that Drosophila might be an excellent model to study interactions between repair pathways.

## 1. Introduction

The comet (single-cell gel electrophoresis) assay is a useful tool to check the presence of DNA damage [1]. Developed around 30 years ago [2] and improved shortly thereafter [3], its use is still growing with different applications in human cells [4,5,6,7,8,9,10] and in other organisms [11,12,13,14,15,16,17]. It has been successfully applied to the model organism *Drosophila melanogaster*, originally using cells from the brain ganglia, although nowadays other cell types are also used [18] (and references therein). This successful application combines the comet assay utilities to study genotoxicity and DNA repair with the Drosophila advantages [18] and the fact that it is an established insect model for human diseases and toxicological research recommended by the European Centre for the Validation of Alternative Methods (ECVAM) [19].

In the analysis of DNA repair, the Drosophila comet assay has been shown to be able to detect differences in DNA damage responses (DDR) among strains mutant for different repair pathways in vivo in somatic cells [20,21,22,23]. In these works, the different responses detected in wild-type and repair mutant strains after treatment of larvae in vivo provided information about the influence of different repair pathways on the removal of specific DNA damage. This approach, the first available approach to check the role of different repair pathways on the DDR in vivo in somatic cells of Drosophila, is considered an in vivo comet assay [24]. 

In mammalian cultured cells, the comet assay has been applied to the analysis of DNA excision repair in vitro in both base (BER) and nucleotide (NER) systems, using cell extracts obtained from cells or tissue samples and measuring their DNA damage incision capacity, with repair enzymes as positive controls [25,26,27]. Basically, in this methodology, substrate DNA containing induced or spontaneous adducts is incubated, after the lysis step, with cell-free protein extracts obtained from cells and/or tissue samples. If the repair enzymes present in the extracts recognize the DNA adducts and start repair processes, they would generate single-strand breaks, at least in the case of excision repair systems [25]. The detection and quantification of these breaks would allow the quantification of DNA repair activities in a new approach called the in vitro comet repair assay, which has been used to study DNA repair in mammalian cells and tissues [26].

Considering the high number of repair mutant strains available, the application of this approach to Drosophila could be a valuable tool to study not only DNA repair activity but also interactions between repair pathways. Moreover, if the cells were to be obtained from in vivo untreated and treated larvae, the approach ex vivo would provide still more relevant information [24].

Therefore, the aim of this work is the implementation of an ex vivo comet assay in Drosophila, checking its usefulness to study and quantitate DDR, and comparing it with the in vivo comet assay in the analysis of possible interactions between DNA repair pathways in these responses. For this purpose, four Drosophila strains, one wild-type efficient repair (*Oregon K*, *OK*), and three repair mutant ones (NER deficient *mus201* [28], protein dmPolQ deficient *mus308* [29], and the double mutant *mus201;mus308* deficient in both systems), were chosen, together with the alkylating agent methyl methanesulfonate (MMS), as a DNA damage inducer [30,31]. The combination of the mutant strains with MMS as the model chemical was chosen for three reasons: (i) to have a known reference to check the ex vivo assay with MMS and the NER system; (ii) to study if dmPolQ processed DNA nitrogen alkylation damage with MMS and the PolQ protein; and (iii) to determine if interactions between DNA repair pathways and/or proteins might be detected with NER and the dmPolQ protein in the response to MMS-induced DNA damage.

The obtained results showed that both approaches detected the role of the NER system and, for the first time, that of the dmPolQ protein on the response to MMS-induced DNA damage in somatic cells and that these two repair pathways acted independently. Moreover, they demonstrated that the ex vivo comet assay allows the relative quantification of repair activities in the repair mutant strains compared to the wild-type ones.

## 2. Material and Methods

### 2.1. Strains

- *Oregon K* (*OK*), a wild-type strain efficient for all the repair systems,
- *mus201*, homozygous for the *mus201^D1^* mutant allele of the *dmXPG* gene [28,32] and, therefore, deficient for the NER repair system
- *mus308*, homozygous for the *mus308^D2^* mutant allele of the gene encoding the dmPolQ protein [29], a homologue of the human DNA polymerase Q [33]. It is deficient in the repair of DNA cross-links and persistent oxygen alkylation DNA damage [34,35], and possibly also in the repair of nitrogen alkylation [36], working both in an alternative end-joining process called microhomology-mediated end joining (MMEJ) and in the damage bypass [37,38].
- *mus201;mus308*, a homozygous double mutant for the *mus201^D1^* and *mus308^D2^* alleles, was obtained in our laboratory by crossing the individual mutant strains using balancer stocks. It lacks both NER and dmPolQ activities.

All of them were maintained at 21 °C (the double mutant strain does not grow at 24 °C), with 12 h light-darkness cycles, and 60% humidity in a standard baker-yeast/sugar medium. 

### 2.2. Chemical

MMS (CAS Nº 66-27-3) was purchased from Sigma-Aldrich, Spain (now MERCK), and dissolved in sterile MilliQ water (18.2 Ω). Fresh solutions were prepared for each experiment. Three different MMS concentrations, 0.1, 0.5, and 1 mM, together with the negative control, were studied in the in vivo assay. Only one MMS concentration, 1 mM, and the negative control were used in the ex vivo comet assay.

### 2.3. Larvae Collection, Treatment, and Cell Isolation

Third-instar larvae were collected from bottles with controlled larvae density: 100 females and 60 males for the wild-type strain and 120 females and 80 males for the mutant strains were allowed to lay eggs for 24 h at 24 °C. After removing the parents, larvae were allowed to develop at 21 °C for 5 additional days. In this way, larvae with 144 ± 12 h of total development were at the third-instar stage and presented a rather large size.

In both comet assay approaches, larvae were treated for 12 h at 24 °C [18] in vials with 3 mL of Carolina instant medium (approx. 0.76 g) hydrated with 3 mL of the chemical solution(s) or the solvent (MilliQ water) in the case of negative controls. A total of 10–15 larvae were treated per vial, and two vials were prepared per analyzed chemical concentration and/or extract.

Immediately after treatment, brain ganglia from 4 larvae per vial were isolated, placed on 30 μL of Ringer solution, and torn apart, as described before [18], to obtain individual cells. 

### 2.4. Extract Preparation

For each strain, extracts were obtained as described before [24]. Briefly, 150 flies (75 females and 75 males) were smashed in 500 µL of cold extraction buffer (45 mM HEPES, 0.4 M KCl, 1 mM EDTA, 0.1 mM DTT, 10% glycerol, pH 8 adjusted with 6 M KOH) with glass pestles, and the solution was maintained at −80 °C in 50 µL aliquots. 

Before using it, 12 µL of 1% Triton X-100 were added to every aliquot, which was then vortexed for 5 s, placed on ice for 5 min, and centrifuged at high speed for 10 min. To the 50 μL supernatant, 200 μL of cold reaction buffer (40 mM HEPES, 0.1 M KCl, 0.5 mM EDTA, 0.2 mg/mL BSA, pH 8 adjusted with 6 M KOH) were added, and they were kept at −20 °C until use. 

To check that protein content was similar in all the extracts, protein concentration was estimated from this mix (supernatant plus reaction buffer) using the Pierce™ BCA^TM^ Protein Assay Kit from Thermo Fisher Scientific™ (Madrid, Spain), following the manufacturer’s instructions.

### 2.5. In Vivo Comet Assay

The comet assay was performed as described in [18]. Briefly, cells from MMS-treated and untreated (negative control) larvae were embedded in 0.5% low melting point (LMP) agarose gels (Invitrogen, Thermo Fisher Scientific, Madrid, Spain) and spread with coverslips on slides previously coated with 150 µL of 0.5% normal melting point (NMP) agarose (Invitrogen). Cells from one vial were used to prepare one gel that was spread on one slide.

Cell lysis was performed with freshly prepared lysis solution (89% lysis buffer (2.5 mM NaCl, 100 mM Na_2_EDTA, 10 mM Tris-(hydroxymethyl)-aminomethane, 0.25 M NaOH; 0.77% N-lauroylsarcosine sodium salt, pH 10), 10% Dimethyl sulfoxide (DMSO), and 1% Triton X-100) at 4 °C for 2 h in darkness. Denaturation was carried out with 1 mM Na_2_EDTA and 300 mM NaOH in pH 12.6 buffer for 20 min at 4 °C in darkness, followed by electrophoresis at 300 mA and 0.9 V/cm for another 20 min at 4 °C in darkness. Gels were neutralized by washing them two times with neutralization buffer (0.4 M Tris-(hydroxymethyl)-aminomethane, pH 7.5), fixed with absolute ethanol for 3 min, and allowed to completely dry overnight at room temperature and darkness.

Before the gel staining with 40 µL of ethidium bromide solution (0.4 µg/mL) and 1 µL of fluorescence protector Vectashield^®^ (Vector Laboratories, Inc., Burlingame, CA 94010, USA), the slides were coded for blind scoring.

### 2.6. Ex Vivo Comet Assay

The comet assay was performed exactly as described above, with one additional step for the incubation with the extracts or their solvent. After the lysis step, the slides were washed twice with cold reaction buffer for 10 min, and placed on a cold surface. Then, the gels were covered with 60 µL of cell extracts or reaction buffer (as an extract negative control) and placed in a humidity chamber at 24 °C for 30 min. Afterwards, the comet assay was resumed with the remaining steps. Two slides from each group (MMS-treated and untreated larvae) were incubated with each extract or reaction buffer (negative control).

### 2.7. Microscope and Image Analysis

The slides were analyzed in an Olympus BX61 fluorescence microscope, equipped with an Olympus DP-70 CCD camera and a 530–560 nm excitation filter, from the Scientific and Technical Services (SCTs) at the University of Oviedo. Photos of at least 50 nucleoids were taken per gel.

Photos were analyzed with the KOMET 5 program (Kinetic Imaging Ltd., now Andor Technology-Oxford Instruments, Oxford, UK), collecting information for the comet parameter tail moment (TM), which is the product of the % tail DNA and tail length divided by 100. In our experience with Drosophila neuroblast cells, this parameter increases linearly with the amount of DNA damage, and it is better than the % tail DNA to detect statistically significant differences [18]. 

### 2.8. Statistical Analysis 

At least three independent experiments were performed for each comet assay. Within individual experiments and due to the observed asymmetry in TM distributions, comparisons between treatments and their respective negative controls were analyzed with the non-parametric statistical Mann–Whitney U-test. Nevertheless, since the average values obtained from the different experiments were normally distributed, their comparisons were analyzed with paired Student *t*-tests. 

In the case of the in vivo comet assay, comparisons between strains were carried out with dose–response linear regression analysis. 

In the case of the ex vivo comet assay, in addition to the comparisons between extracts carried out with paired Student *t*-tests, the relative repair activity (or incision activity) of each mutant strain with respect to that of the wild-type efficient repair strain was estimated as described before [24]:

Relative activity on spontaneous DNA damage (%) = (MRBC-BC/ERBC-BC) × 100

Relative activity on induced DNA damage (%) = (MRTC-TC/ERTC-TC) *×* 100

where *BC* and *TC* are the results of cells treated with the negative control and 1 mM MMS, respectively, and incubated with the reaction buffer, whereas *MRBC* and *MRTC*, and *ERBC* and *ERTC*, are the results of these same cells incubated with mutant repair extracts (*MR*) or efficient repair extracts (*ER*), respectively.

### 2.9. Interaction between Repair Systems

The possible interaction between the NER system and the dmPolQ protein in the response to MMS-induced DNA damage, both in vivo and ex vivo, was studied through the estimation of an Interaction Factor (IF), as described by Katsifis et al. [39], with modifications:

IF = (Damage induced in *OK* strain + damage induced in the *mus201;mus308* strain) − (damage induced in *mus201* strain + damage induced in *mus308* strain)

The standard error (SE) of this IF was estimated, with the standard errors of the negative controls (NC) and of the MMS treatments (MMS) for every strain, as follows:

SE (IF) = √ (SE NC *OK* strain)^2^ + (SE MMS *OK* strain)^2^ + (SE NC *mus201;mus308* strain)^2^ + (SE MMS *mus201;mus308* strain)^2^ + (SE NC *mus201* strain)^2^ + (SE MMS *mus201* strain)^2^ + (SE NC *mus308* strain)^2^ + (SE MMS *mus308* strain)^2^

A zero value in this factor indicates additive effects and, therefore, independence between the analyzed pathways. Values different from zero indicate interactions between them, either positive (synergism) or negative (antagonism).

## 3. Results and Discussion

### 3.1. In Vivo Comet Assay

The in vivo comet assay was carried out by treating larvae from the different strains in vivo with three MMS concentrations and the negative control for 12 h and performing the comet assay with neuroblast cells. No toxicity analysis was performed in this assay because no death larvae, from any strain, were found after the treatment with the different MMS concentrations. However, the presence of “hedgehog” comets in the gels corresponding to the two highest MMS concentrations in the mutant strains (especially in the double mutant one) and not detected in the wild-type strain could indicate a certain cell toxicity [40] with respect to the respective negative controls. 

A higher sensitivity to the toxic effects of MMS was expected for the *mus201* strain [41] and, therefore, also for the double mutant *mus201;mus308* one; a higher sensitivity to nitrogen alkylation DNA damage could also be expected for the *mus308* strain, at least when the levels of DNA damage are high [36]. 

The average TM values obtained for each analyzed strain are presented in Table 1. 

As observed, there were differences in the spontaneous levels of DNA strand breaks among strains, with the highest value in the wild-type strain and the lowest in the double mutant one. Although one possible explanation might be that in efficient repair strains more breaks should be detected simply because of the activity of the excision repair systems, another explanation, which adds significantly, would be the different genetic background of the strains, which listed *OK* strains as highly sensitive to different chemicals [18,42].

**Figure 1:**
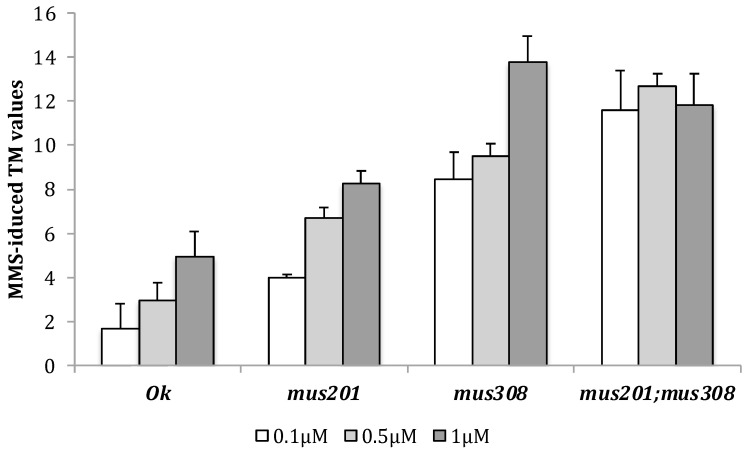
MMS-induced DNA damage in vivo. Arithmetic means and standard errors of MMS-induced TM values, from at least three independent experiments, for all the analyzed concentrations in each studied strain.

Because of this, only the MMS effect should be compared among strains; clearly, this chemically induced DNA strand breaks in all the strains in a linear relationship with the dose in all of them, but in the double mutant one, as shown in Figure 1, where the averaged MMS-induced TM values obtained for each analyzed strain are presented. 

MMS was chosen as a genotoxic agent because it induces mostly nitrogen alkylations [30], some of which can indirectly induce DNA strand breaks through the generation of abasic (AP) sites [31], and because nitrogen alkylations are repaired in Drosophila mainly by the NER system [40], since no methylpurine DNA glycosylase has ever been found [43]; moreover, the dmPolQ protein seems to play a role in the processing of nitrogen ethylations in germ cells, especially when NER is saturated by high levels of DNA damage [36]. Therefore, these results fit with the known mechanism of action for this chemical.

To compare the responses from the different strains, regression analyses were performed for each strain, and the obtained equations, as well as their fits, are presented in Table 2. Comparisons revealed that there were no differences in slope among the strains, but there were statistically significant differences in elevations among all the strains, with the exception of mus308 and the double-mutant strain.

These results indicate that the level of induced DNA damage increased in the different strains in the order *Ok* < *mus201* < *mus308* = *mus201;mus308*. The response in the double-mutant strain, with no increase with concentration, might be due to the mentioned cell toxicity. 

According to these results, the in vivo comet assay detected differences in DNA damage responses between the wild-type and the repair mutant strains. Thus, this assay detected an effect of the NER system on the response to MMS-induced DNA damage, confirming previous data [20]: an active NER system contributes to generating strand breaks, but when it is not working, strand breaks are still produced because the levels of adducts forming AP sites and/or blocking DNA replication would be much higher. 

In addition to the NER effect, this assay also detected an effect of the dmPolQ protein, which is a DNA polymerase involved in DNA damage bypass [20,34,35,38] and in the MMEJ process [37,38]. When this protein is not working, DNA strand breaks could be formed from MMS-induced DNA damage if this damage were not bypassed, and, moreover, once formed, the breaks would not be re-joined. To our knowledge, this is the first time that this effect has been detected in somatic cells, and it agrees with that detected in Drosophila germ cells in vivo [36]. 

To study possible interactions between the NER system and the dmPolQ protein, Interaction Factors were estimated with the results of 0.1 and 0.5mM MMS concentrations. The obtained values, 0.82 (with 95% CI, −4.27, 5.91) for 0.1 mM and −0.58 (with 95% CI, −3.58, 2.42) for 0.5 mM, none of them different from zero, indicated that these two repair pathways contributed additively, that is, independently, to the response (repair and/or processing) to MMS-induced DNA damage.

### 3.2. Ex Vivo Comet Assay

In this assay, larvae from the OK strain were treated in vivo with 1 mM MMS (MMS treated) or with MilliQ (MMS untreated). In each experiment, 10 slides were prepared with cells from MMS-treated larvae and 10 slides with cells from MMS-untreated ones. These slides were incubated ex vivo with the reaction buffer (Buffer) or with extracts from the *OK*, *mus201*, *mus308*, and *mus201;mus308* strains at two slides per extract/buffer. 

As indicated, protein content in the strain extracts was checked with at least three measures from different extract aliquots for each strain after the addition of the reaction buffer. Results were 2.32 ± 0.08 μg/μL protein for the *mus308* strain, 2.49 ± 0.21 μg/μL for the *OK* strain, 2.55 ± 0.11 μg/μL for the double mutant *mus201;mus308* strain, and 2.83 ± 0.16 μg/μL for the *mus201* strain. Since the contents were rather similar among strains, the TM data were adjusted for protein content.

**Figure 2:**
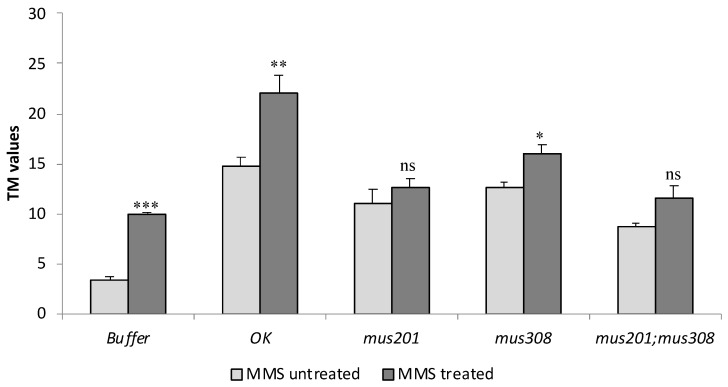
Ex vivo DNA damage response analysis. Mean TM values, adjusted for protein content, and their SE (*n* = 3), induced by the ex vivo incubation with cell-free extracts from all the analysed strains and with their solvent (Buffer), on nucleoids from *OK* larvae treated in vivo with 1 mM MMS (MMS treated) or with MilliQ water (MMS untreated). Asterisks indicate comparisons between MMS treated and untreated nucleoids for each extract: * *p* < 0.05; ** *p* < 0.01; *** *p* < 0.001; ns, non-significant.

These results are presented in Figure 2. It is clear that for all the extract/buffer, the detected DNA damage was higher in the MMS-treated cells than in the MMS-untreated cells, although for *mus201* and *mus201;mus308*, the differences were not statistically significant. 

They indicated, first of all, that MMS had induced DNA strand breaks in vivo. Secondly, they indicate that in *Ok* and *mus308* extracts, there were proteins that detected and incised MMS-induced DNA damage. Finally, the fact that extracts from *mus201* and *mus201;mus308* strains did not generate additional DNA strand breaks demonstrated that no detectable active incision proteins were present in them. 

Assuming that the strand breaks generated with the ex vivo extract incubation were due to the incision activity of repair pathways [24], then the fact that ex vivo incubations with any extract increased the level of DNA strand breaks on MMS-untreated cells, compared to the buffer, demonstrates that, even although we treated efficient repair *OK* larvae, there still was non-repaired DNA damage in the analyzed cells, and that the extracts from all the strains were detecting and processing spontaneous DNA damage; however, only those from *OK* and *mus308* strains were doing the same on MMS-induced DNA damage. These results demonstrated again that there was non-repaired MMS-induced DNA damage in the wild-type cells and that mus201 strains lack incision activity over the induced damage, as expected from dmXPG mutants [28,32], and that the NER system is the most relevant repair pathway for the detection and removal of DNA methylations induced by this chemical in somatic cells, as already demonstrated for germ cells [41]. 

When the effects of extracts from each repair mutant strain were compared to those of the *OK* strain, results revealed that all of them induced fewer DNA strand breaks, both in MMS-treated and untreated cells, but differences were not statistically significant for *mus308* extracts in MMS-untreated cells. 

Possible interactions between the NER system and the dmPolQ protein were also estimated with this ex vivo assay, obtaining an IF value of 5.00 with a 95% CI (−0.05, 10.05), not statistically significantly different from zero. This result, as well as those obtained with the in vivo assay, demonstrate independence between the NER pathway and the dmPolQ protein in the response to MMS-induced DNA damage. 

**Figure 3:**
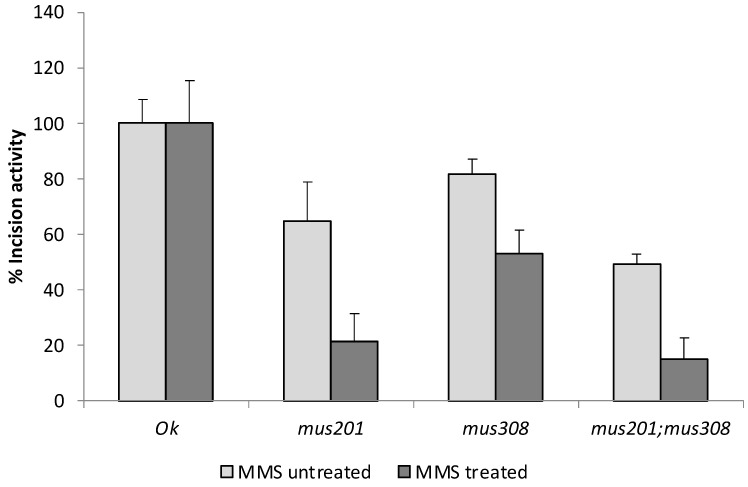
Incision activity of the analyzed strains. Relative averaged incision activities of the repair mutant strains, and their standard errors (*n* = 3), compared to the activity of the OK strain, which is considered 100%.

The relative DNA-damaged incision activities of the mutant strain extracts, compared to those of the *OK* wild-type one, estimated as described in Section 2.9, with the averaged induced DNA strand breaks from every strain, are presented, with their respective standard errors, in Figure 3. 

As indicated by Gaivão et al. [24], repair mutant strains should be compared to the repair-efficient one over the same level of DNA damage, and, therefore, relative activities were estimated separately for MMS-untreated and MMS-treated cells. For spontaneous DNA damage (MMS-untreated cells), the relative incision activities of each mutant strain were 64.9%, 81.7%, and 49.2% for *mus201*, *mus308*, and *mus201;mus308*, respectively. 

For MMS-induced damage, the relative incision activities of the repair mutant strains were 21.5%, 52.9%, and 14.8% for *mus201*, *mus308*, and *mus201;mus308*, respectively.

These results demonstrated a clear effect, first of the NER system on the detection of both spontaneous and MMS-induced DNA damage, and second of the dmPolQ protein on the processing of MMS-induced but not spontaneous DNA damage. In agreement with these results, data on germ cells demonstrated the effect of NER on the repair of both spontaneous [44,45,46] and MMS-induced damage [41,47], the lack of effect of the dmPolQ protein on spontaneous DNA damage [34,35,36], and the possible role of this protein in the processing of nitrogen alkylation damage [36]. 

Considering the incubation with the double mutant *mus201;mus308* strain extract, results indicated differences with the *OK* extract and also with the *mus308* one, but not with the *mus201* extract, for MMS-treated and untreated cells, confirming a more important role for NER than for the dmPolQ protein on the response to DNA damage.

## 4. Concluding Remarks

The results of this work confirm the importance of the Drosophila comet assay to study DNA damage response. (i) They demonstrate that the in vivo comet assay allows the analysis of DDR in vivo in somatic cells of Drosophila and that it is a valuable tool in the deciphering of interactions between repair mechanisms or pathways, as in the case of the dmPolQ protein and its lack of interaction with the NER pathway in the response to MMS-induced DNA damage. (ii) Considering the described problems in analyzing the effects of the dmPolQ protein in vivo, different from those detected in vitro, due to possible interactions with other repair proteins [38], the use of the comet assay, both in vivo and ex vivo, as presented here, could be a useful and powerful tool to find interactions between proteins and, therefore, to discover hidden effects. (iii) These results also demonstrate that, in addition to detecting interactions between strains and proteins, the ex vivo comet assay may provide information on repair mutant strains, like their role in DNA strand break generation, that is, their incision activities, and, more importantly, it allows the quantification of their DDR. (iv) Moreover, since the analyzed cells were neuroblasts from third-instar larvae, these results demonstrate that the studied repair pathways are active in brain cells throughout the development stages.

Finally, it is necessary to remember the usefulness of *D. melanogaster* as a model organism since its genome shares a high homology with the human one, contains orthologs to most human genes associated with genetic diseases [48,49], its xenobiotic metabolism is homologous to that of humans, it helps to study chemotherapy responses [50], and there is a very high homology between its DNA repair pathways and those of humans [51]. Because of this, the additive effect of the dmPolQ protein and the NER pathway on the response to MMS-induced DNA damage might be extrapolated to humans. Moreover, Drosophila has been used in the last few years to study different processes relevant to humans, like regeneration [52], nutrition research [53], neurobiology and neurodegenerative diseases [48,54], and cancer [55,56], among others, thanks to the existence of many mutant strains for many different genes and lately because mutant strains may be created on demand to model human-specific mutations or mutation combinations, especially related to cancer [57,58]. Since in some of these processes DNA damage plays a fundamental role, both in the development of diseases as well as in their treatments, the use of the Drosophila comet assay, as described here with neuroblast cells, either in vivo or ex vivo, may provide relevant data about protein interactions and, therefore, useful information for patient handling.

## Figures and Tables

**Table 1:** Comet tail moment values obtained after MMS in vivo treatment of larvae from the analyzed strains. Numbers are arithmetic means of at least three independent experiments, and their standard errors. Asterisks indicate differences between treatments and their corresponding negative controls.

	Strains			
Concentration (mM)	*OK*	*mus201*	*mus308*	*mus201;mus308*
0	6.20 ± 0.45	4.70 ± 0.57	3.29 ± 0.53	2.61 ± 0.14
0.1	7.89 ± 1.12	8.71 ± 0.12 **	11.76 ± 1.22 **	14.21 ± 1.78 **
0.5	9.16 ± 0.79 **	11.42 ± 0.48 ***	12.79 ± 0.55 ***	15.28 ± 0.60 ***
1	11.13 ± 1.17 **	12.98 ± 0.58 ***	17.08 ± 1.16 ***	14.43 ± 1.42 **

** *p* < 0.01; *** *p* < 0.001.

**Table 2:** Linear dose–response regression analyses of MMS-induced DNA damage. Regression equations and their fits for each studied strain. * *p* < 0.05; ** *p* < 0.01; *** *p* < 0.001; ns, non-significant, comparing each slope with slope 0.

Strain	Equation	R^2^	Slope
*Ok*	y = 3.61x + 1.27	0.996	3.61 ± 0.22 *
*mus201*	y = 4.68x + 3.84	0.953	4.68 ± 0.75 ***
*mus308*	y = 6.02x + 7.37	0.926	6.02 ± 1.61 **
*mus201;mus308*	y = 0.16x + 11.95	0.017	0.16 ± 1.24 ^ns^

## Data Availability

Data are available at the Corresponding author.
